# Effects of chloride content of intravenous crystalloid solutions in critically ill adult patients: a meta-analysis with trial sequential analysis of randomized trials

**DOI:** 10.1186/s13613-019-0506-y

**Published:** 2019-02-13

**Authors:** Ming Xue, Xiwen Zhang, Feng Liu, Wei Chang, Jianfeng Xie, Jingyuan Xu, Yi Yang, Haibo Qiu

**Affiliations:** Department of Critical Care Medicine, Zhongda Hospital, School of Medicine, Southeast University, Nanjing, 210009 China

**Keywords:** Lactated Ringers, Plasma-Lyte 148, 0.9% Saline, Critically ill patients, In-hospital mortality, Renal outcome

## Abstract

**Background:**

Intravenous crystalloid solutions are administered commonly for critically ill patients. We performed this meta-analysis of randomized trials with trial sequential analysis (TSA) to evaluate effects of chloride content of intravenous crystalloid solutions on clinical outcomes among critically ill adult patients.

**Methods:**

Electronic databases were searched up to June 1, 2018, for randomized trials of use of balanced crystalloids versus 0.9% saline solutions in critically ill adult patients. The outcome variables included mortality, renal outcomes, serum content alterations and organ function. Subgroup analysis was conducted according to patient settings, types or volume of crystalloid fluid, or among sepsis versus non-sepsis, TBI versus non-TBI or subpopulations by the categories of baseline kidney function. Random errors were evaluated by trial sequential analysis.

**Results:**

Eight studies with 19,301 patients were analyzed. A trend of in-hospital survival benefit with no statistical difference could be observed with balanced crystalloids compared with 0.9% saline (RR 0.92, 95% CI 0.85–1.0, *p* = 0.06). The use of balanced crystalloid solutions was associated with longer RRT-free days (SMD 0.09, 95% CI 0.06–0.12, *p* < 0.001), less risk of increase in serum concentrations of chloride (SMD − 1.23, 95% CI − 1.59 to − 0.87, *p* < 0.001) and sodium (SMD − 1.28, 95% CI − 1.65 to − 0.92, *p* < 0.001), less risk of decline in serum base deficit (SMD − 0.58, 95% CI − 0.98 to − 0.18, *p* = 0.004), longer ventilator-free days (SMD 0.08, 95% CI 0.05–0.11, *p* < 0.001) and vasopressor-free days (SMD 0.04, 95% CI 0.00–0.07, *p* = 0.02). Subgroup analysis showed that balanced crystalloid solutions were associated with a reduced in-hospital mortality rate among septic patients (RR 0.86, 95% CI 0.75–0.98; *p* = 0.02) and non-traumatic brain injury patients (RR 0.90, 95% CI 0.82–0.99, *p* = 0.02), while the TSA results indicated a larger sample size is still in need.

**Conclusions:**

Limited evidence supported statistical survival benefit with balanced crystalloid solutions, while it benefited in reducing organ support duration and fluctuations in serum electrolyte and base excess and was associated with decreased in-hospital mortality in subpopulation with sepsis and non-TBI. Large-scale rigorous randomized trials with better designs are needed to provide robust evidence for clinical management.

*Trial registration* The protocol for this meta-analysis was registered on PROSPERO: International prospective register of systematic reviews (CRD42018102661), https://www.crd.york.ac.uk/prospero/#recordDetails

**Electronic supplementary material:**

The online version of this article (10.1186/s13613-019-0506-y) contains supplementary material, which is available to authorized users.

## Background

Intravenous crystalloid solutions are administered commonly in critical care unit, especially for patients with need of resuscitation. Crystalloid solutions as the fluid of choice for initial resuscitation and subsequent intravascular volume replacement in patients with sepsis and septic shock were recommended in guidelines for management of sepsis and septic shock published in 2016 [1]. Resuscitation with large volumes of crystalloid solutions with non-physiological content may lead to electrolyte disturbance and hyperchloremic metabolic acidosis which could result in severe renal, cardiac or hepatic disease. Different component electrolytes of the crystalloid solutions interact with the body’s internal equilibrium [2].

0.9% saline solutions have been the most commonly administered intravenous fluid. Since first developed by adding the buffer lactate to Ringer’s solution in the 19th century, balanced crystalloid solutions such as (Plasma-Lyte 148, lactated or acetate Ringer) have been preferable alternative for relatively physiological concentrations of ions [3–5]. Data suggest that 0.9% saline can increase the risk of hyperchloremic metabolic acidosis and related complications, such as acute kidney injury: One before and after prospective trial has shown the beneficial effects of chloride-restrictive intravenous fluid in the improvement of preventing acute kidney injury (AKI) and decreasing use of renal replacement therapy (RRT) in critically ill patients [6]. In addition, saline administration has been proved to result in reductions in renal blood flow velocity and renal cortical tissue perfusion following sustained hyperchloremia compared with Plasma-Lyte 148 in healthy adults [7]. For critically ill patients, it should be more cautious when choosing crystalloid fluid type for initial resuscitation and subsequent intravascular volume maintenance. However, 2016 Surviving Sepsis Campaign (SSC) guidelines could not provide recommendations for the use between normal saline of supraphysiological chloride content and balanced salt solution of low chloride content with indirect low-quality evidence from observational or retrospective studies [1, 8, 9].

A recent large pragmatic trial suggested balanced crystalloid fluid administration in critically ill adults compared with use of 0.9% saline could decrease incidence of a major adverse kidney event within 30 days [10] rather than mortality or other renal-related outcomes. Effects of chloride content of intravenous crystalloid solutions in critically ill patients were still controversial. Therefore, the objective of this meta-analysis with trial sequential analysis (TSA) was to evaluate effects of balanced crystalloids compared to controls with 0.9% saline on mortality and renal outcomes for critically ill adult patients, with which minor differences might be detected in patient outcomes with statistical power, providing the hint for clinical practice.

## Methods

### Approval

There is no requirement for ethical approval, and patient consent for this meta-analysis is based on previous published studies. The protocol for this meta-analysis was registered on PROSPERO: International prospective register of systematic reviews (CRD42018102661).

### Search strategy and study selection

We conducted a search for randomized trials in the following databases until June 2018: Medline, EMBASE, Cochrane (Central) database, Elsevier, Web of Science and ClinicalTrials.gov. The details of search strategy were as the meta-analysis by Kawano-Dourado used [11], shown in Additional file 1. There was no language restriction.

The titles and abstracts were screened to determine whether a study should be included by two reviewers (MX and FL) independently. Then, the full texts were reviewed according to the inclusion and exclusion criteria. Any discrepancies were resolved by a consensus on the inclusion or exclusion of a study after a discussion with a third reviewer.

### Inclusion and exclusion criteria

We included trials with the following features:

1. Type of study: randomized controlled trials and cluster randomized trials.
2. Population: Acutely ill adult patients in the ICU or surgical adult patients transferred to ICU in the perioperative period.
3. Intervention: balanced crystalloid fluid characterized by a near-physiological chloride concentration (ion concentration 111 mmol/l or less, including Plasma-Lyte 148, lactated or acetate Ringers), given intravenously for resuscitation or maintenance.
4. Control: 0.9% saline solution works as control group with relatively high chloride content for fluid resuscitation or maintenance.
5. Outcomes: The primary outcome was in-hospital mortality. The secondary outcomes were renal outcomes including new RRT use, stage 2 or higher AKI development according to kidney disease: improving global outcomes (KDIGO) criterion [12] after enrollment, RRT-free days and the incidence of MAKE30 [10]; the change of serum content concentrations including chloride, sodium, pH value, bicarbonate and base acid; mechanical ventilation (MV) use, MV-free days and vasopressor-free days.

We excluded trials with the following features:

1. If they made the comparisons between crystalloids versus colloids, hypertonic saline versus balanced solutions, or among solutions with different colloid components, to minimize the confounding factors and make sure the difference between the experimental and control groups involved a buffer in the solution (usually lactate and/or acetate).
2. If they were involved with the patients receiving renal transplantation.

### Data extraction and synthesis

All available data including characteristics of the selected studies, details of the population enrolled, details of the intervention including type of crystalloid fluid, volume and duration and details of the predefined primary outcome were independently extracted by two investigators (MX and FL). Disagreements between the two investigators were resolved by a consensus after discussing with a third reviewer (XZ).

### Quality assessment

We summarized the evidence by evaluating design, quality, consistency, precision, directness and possible publication bias of the included studies in accordance with the Grading of Recommendations Assessment, Development and Evaluation (GRADE) system (high, moderate, low and very low) [13].

### Statistical analysis

Data were conducted by RevMan 5.3 software according to the Cochrane Handbook. Mantel–Haenszel (M–H) Chi-square test and the *I*^2^ test were used to assess the statistical heterogeneity and inconsistency in RevMan 5.3 [14]. A *p* value < 0.10 was predefined as the statistically significant heterogeneity in the M–H Chi-square test, while *I*^2^ index was used to assess heterogeneity in the meta-analysis. Higgins and colleagues proposed 25%, 50% and 75% of *I*^2^ values would mean low, medium and high heterogeneity, respectively [14]. Both the fixed and random models were used to report the data. A risk ratio (RR) with 95% confidence interval (CI) for the dichotomous data and standardized mean differences (SMD) with 95% CI for the continuous data were reported. The visual inspection of the funnel plot was established to evaluate the publication bias by RevMan 5.3 software (The Nordic Cochrane Centre, Rigshospitalet, Copenhagen, Denmark) according to the Cochrane Handbook [15].

Predefined subgroup analysis was conducted comparing patients setting (ICU versus transferred to ICU in perioperative period), crystalloid fluid type (lactated Ringers, Plasma-Lyte or mixed of both) and fluid volume (mean or median volume above versus < 5 L). To investigate the potential sources of heterogeneity, we performed post hoc analyses according to specific patients. Patients were classified into sepsis and non-sepsis, TBI and non-TBI or subpopulations according to the categories of baseline kidney function by Semler study in 2018 [10]: normal kidney function, AKI, chronic kidney disease (CKD) and RRT prior to enrollment. To better explore the effects of SMART and SPLIT trials with large sample size on our study, we perform sensitivity analysis by removing each single study and additional subgroup analysis by sample size and risk levels of bias.

### Additional analysis

Sensitivity analyses were used to estimate the effect of chloride content of intravenous fluid on clinical outcomes of in-hospital, 30-day and 60-day mortality, development of stage 2 or higher AKI, new RRT use, RRT-free days. It was conducted by sequentially omitting a single study each time, to identify the potential influence of an individual study by STATA (Stata Corporation, College Station, TX, USA).

To prevent the risk of random error from being increased by repeated updates, a two-sided trial sequential analysis (TSA; TSA software version 0.9 Beta; Copenhagen Trial Unit, Copenhagen, Denmark) was performed with *α* = 0.05 and *β* = 0.10 (power 90%) and a required diversity-adjusted information size based on the intervention effect suggested by the included trials using a random-effects model, which aimed to assess the effects of chloride content of intravenous crystalloid fluid on in-hospital mortality of critically ill patients [16–19].

## Results

### Summary of the randomized trial characteristics

**Fig. 1:**
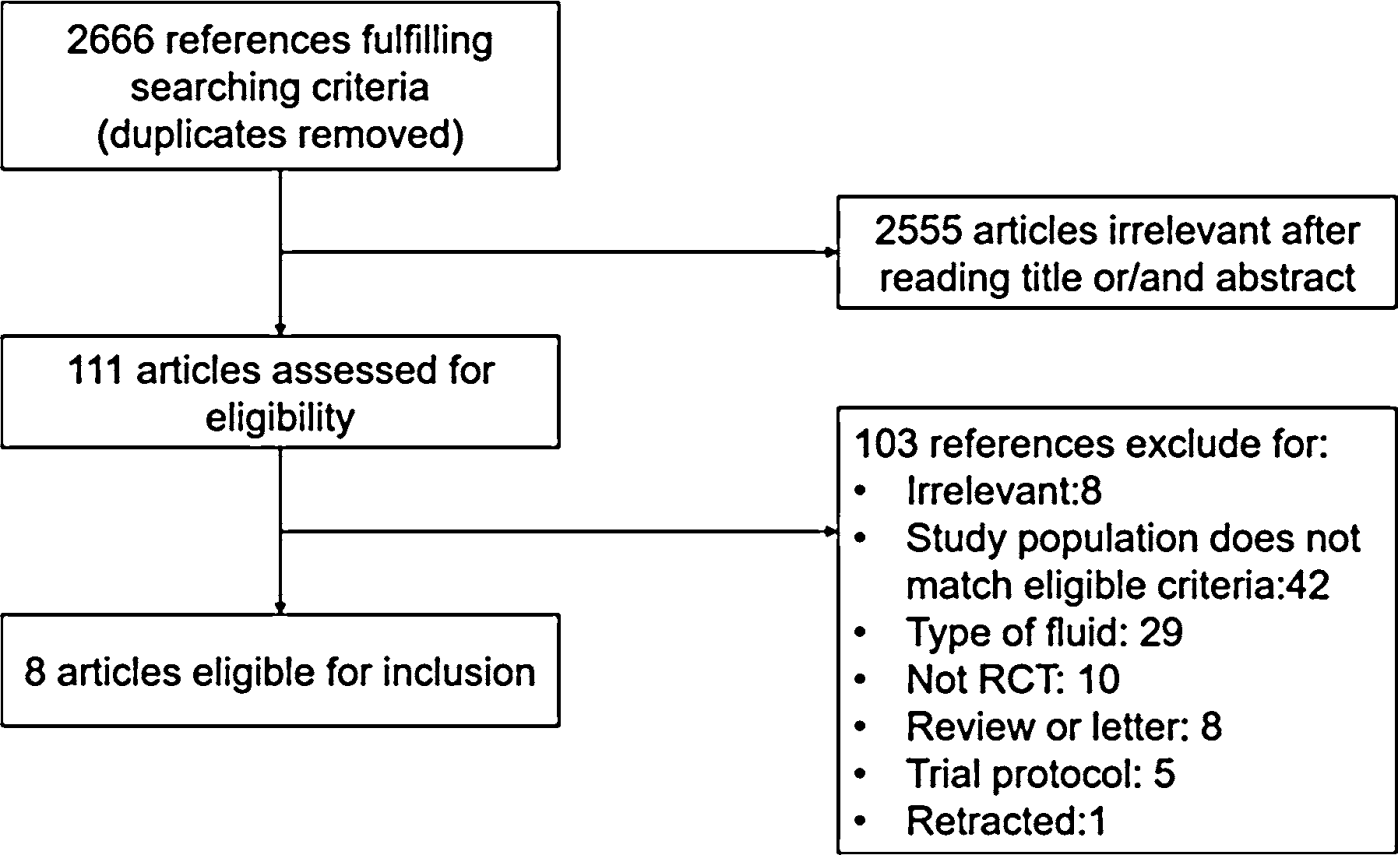
Flow diagram of trial selection

The flow diagram shows the study selection process in Fig. 1. Overall, we identified 2666 articles and excluded 2555 after screening the titles and abstracts for the terms “saline,” “balanced solution,” “randomized control trial” and “critically ill.” Eight trials with 19,326 patients were finally included in this meta-analysis after retrieving 103 full-length manuscripts. The subjects who were included were critically ill adult patients and were randomized to buffered crystalloids of low chloride content versus saline administration. A total of 9744 subjects received balanced solutions, while the remainder were in the control group. Among all the included studies, six studies followed the patients until hospital discharge [9, 20–24], and the others followed the patients for 60 days [10, 25]. Ninety-six (0.5%) patients of two studies included were transferred to ICU postoperation [20, 21], while most of studies were performed in the ICU setting [9, 10, 22–25]. The main characteristics of the included studies are shown in Table 1.

**Table 1:** Characteristics of the included studies

References	No. of patients (analyzed/included)	Setting	Balanced crystalloid fluid type	Follow-up period	Volume of fluid in liters, mean ± SD, median (IQR)	Outcomes		
						Report on mortality	Report on new RRT use	Report on development of stage 2 or higher stage AKI
Waters et al. [20]	66/66	Perioperative transferred to ICU: abdominal aortic aneurysm repair	Lactated Ringers (LR)	In-hospital	Balanced: 6.9 (5.7–7.9) 0.9% saline: 7.0 (5.0–8.5)	√	×	√
Takil et al. [21]	30/30	Perioperative transferred to ICU: major spine surgery	LR	In-hospital	Balanced: 5.1 ± 0.9 0.9% saline: 5.1 ± 1.5	√	√	×
Van Zyl et al. [22]	54/57	Critically ill patients: diabetic ketoacidosis	LR	In-hospital	NR	√	×	×
Young et al. [23]	46/65	Critically ill patients: trauma	Plasma-Lyte	In-hospital at day 30	Balanced:10.3 ± 6.5 0.9% saline: 9.0 ± 5.5	√	×	√
Young et al. [9]	2262/2262	Critically ill patients: mixed	Plasma-Lyte	In-hospital	Balanced: 2.0 (1.0–3.5) 0.9% saline: 2.0 (1.0–3.2)	√	√	√
Verma et al. [24]	67/70	Critically ill patients: mixed	Plasma-Lyte	In-hospital	Balanced: 2.9 0.9% saline: 3.4	√	√	√
Semler et al. [25]	974/974	Critically ill patients: mixed	LR or Plasma-Lyte	60 days	Balanced: 1.6 (0.5–3.6) 0.9% saline: 1.4 (0.5–3.4)	√	√	√
Semler et al. [10]	15,802/15,802	Critically ill patients: mixed	LR or Plasma-Lyte	60 days	Balanced: 1.0 (0–3.0) 0.9% saline: 1.0 (0–3.0)	√	√	√

*No.* number, *SD* standard difference, *IQR* interquartile range, *RRT* renal replacement therapy, *AKI* acute kidney injury according to KDIGO criterion, *LR* lactated Ringers, *NR* no report

### Fluid interventions

A total of 19,301 patients reporting outcomes and analyzed were exposed to crystalloid solutions of different chloride contents as outlined in Table 1. Lactated Ringers alone as balanced crystalloid solution exposures was received in experimental group arms by 48 patients [20–22] and Plasma-Lyte 148 alone by 1207 patients [9, 23, 24]. The majority of the studies, including the two largest studies included [10, 25] used a mixed low-chloride crystalloid solution of lactated Ringers and Plasma-Lyte 148. Only 142 subjects (8.3%) in our meta-analysis received volume with a median or mean around 5L or above [20, 21, 23]. Most of the patients (19,105/19,301) experienced a relatively low, 2–3 L study fluid exposure during the follow-up period [9, 10, 24, 25]. One study did not report the crystalloid fluid volume [22].

### Risk of bias and GRADE levels

We assessed each included trial by the mode of randomization, allocation concealment, level of blinding and loss to follow up (Additional file 1: Figure S1). Four studies [9, 20, 22, 24] were judged to be at low risk of bias, with adequate randomized sequences, concealed allocation and analyzed outcomes of patients by assigned group. Three trials were judged to be at high risk of bias due to performance bias [10, 25] or attrition bias [23]. Risk of bias was unclear for the remaining one study [21]. The GRADE profile shows that evidence of this meta-analysis is of low quality for in-hospital mortality and of low or very low quality for renal outcomes, as seen in Additional file 1: Figure S2, suggesting further studies might be needed to reach a solid conclusion.

### Impact on mortality

**Fig. 2:**
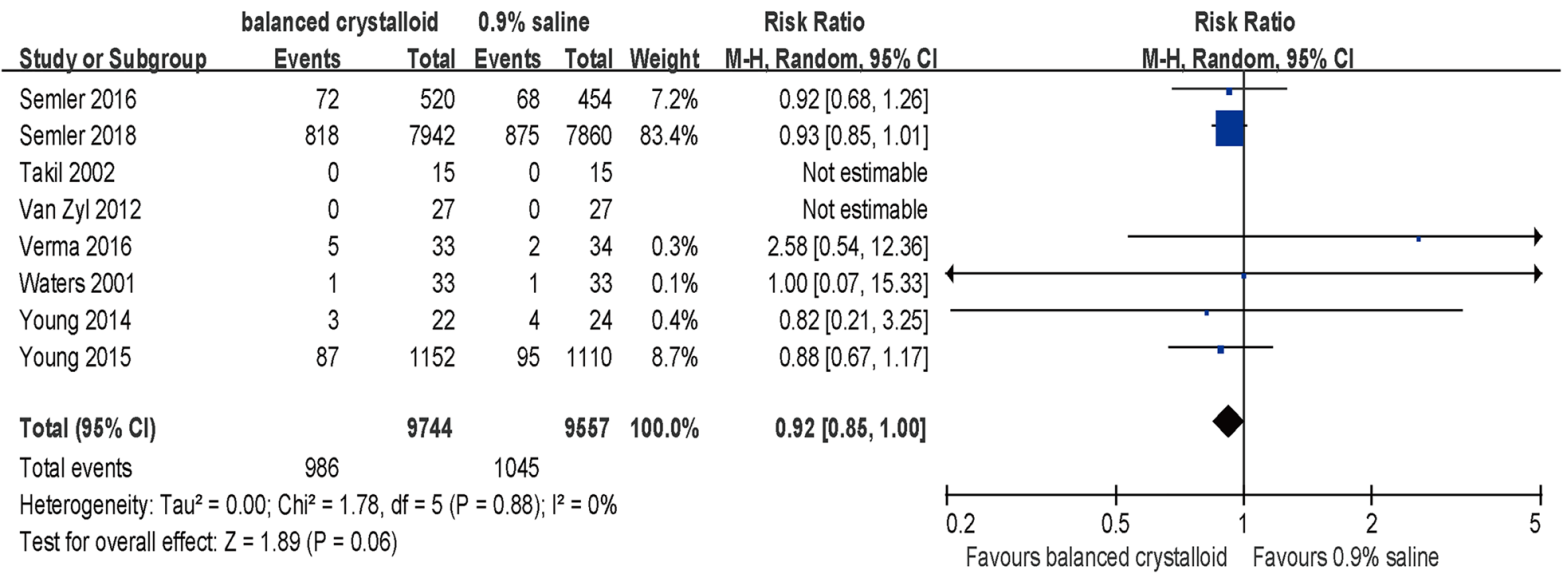
Forest plots for in-hospital mortality of overall population

Among the included studies, 19,301 patients reported the in-hospital mortality and were included in the primary analysis, among which the pooled in-hospital mortality rate was 10.1% (986/9744) and 10.9% (1045/9557) in groups of balanced crystalloids and 0.9% saline, respectively. Pooled estimates of included studies indicated a trend toward survival benefit with no significant difference in patients receiving balanced crystalloid solutions versus 0.9% saline with an RR of 0.92 (95% CI, 0.85–1.00; *p* = 0.06, Fig. 2). There was no significant heterogeneity (*p* = 0.88, *I*^2^ = 0%) among all in-hospital mortality analyses (Table 2). Meanwhile, there were no significant differences in the 30-day and 60-day mortality between groups with balanced crystalloids and 0.9% saline (Table 2).

**Table 2:** Effects of balanced crystalloids versus 0.9% saline fluid on mortality and renal outcomes

Outcomes	Balanced crystalloids	0.9% Saline	Risk ratio/standard mean difference (95% CI)	
In-hospital mortality	Events/total	Events/total	Fixed models	Random models
Waters et al. [20]	1/33	1/33	1.00 [0.07, 15.33]	1.00 [0.07, 15.33]
Takil et al. [21]	0/15	0/15	Not estimable	Not estimable
Van Zyl et al. [22]	0/27	0/27	Not estimable	Not estimable
Young et al. [23]	3/22	4/24	0.82 [0.21, 3.25]	0.82 [0.21, 3.25]
Young et al. [9]	87/1152	95/1110	0.88 [0.67, 1.17]	0.88 [0.67, 1.17]
Verma et al. [24]	5/33	2/34	2.58 [0.54, 12.36]	2.58 [0.54, 12.36]
Semler et al. [25]	72/520	68/454	0.92 [0.68, 1.26]	0.92 [0.68, 1.26]
Semler et al. [10]	818/7942	875/7860	0.93 [0.85, 1.01]	0.93 [0.85, 1.01]
Summary	986/9744	1045/9557	0.92 [0.85, 1.00]	0.92 [0.85, 1.00]
Fixed model heterogeneity: *χ*^2^ = 1.78, *df* = 5 (*p* = 0.88); *I*^2^ = 0%; test for overall effect: *Z* = 1.88 (*p* = 0.06)				
Random model heterogeneity: *τ*^2^ = 0.00; *χ*^2^ = 1.78, *df* = 5 (*p* = 0.88); *I*^2^ = 0%; test for overall effect: *Z* = 1.89 (*p* = 0.06)				

Outcomes	Balanced crystalloids	0.9% Saline	Risk ratio/standard mean difference (95% CI)	
30-day mortality	Events/total	Events/total	Fixed models	Random models
Young et al. [23]	3/22	4/24	0.82 [0.21, 3.25]	0.82 [0.21, 3.25]
Semler et al. [25]	72/520	68/454	0.92 [0.68, 1.26]	0.92 [0.68, 1.26]
Semler et al. [10]	818/7942	875/7860	0.93 [0.85, 1.01]	0.93 [0.85, 1.01]
Summary	893/8484	947/8338	0.92 [0.85, 1.01]	0.92 [0.85, 1.01]
Fixed model heterogeneity: *χ*^2^ = 0.03, *df* = 2 (*p* = 0.98); *I*^2^ = 0%; test for overall effect: *Z* = 1.78 (*p* = 0.08)				
Random model heterogeneity: *τ*^2^ = 0.00; *χ*^2^ = 0.03, *df* = 2 (*p* = 0.98); *I*^2^ = 0%; test for overall effect: *Z* = 1.78 (*p* = 0.08)				

Outcomes	Balanced crystalloids	0.9% Saline	Risk ratio/standard mean difference (95% CI)	
60-day mortality	Events/total	Events/total	Fixed models	Random models
Semler et al. [25]	87/520	83/454	0.92 [0.70, 1.20]	0.92 [0.70, 1.20]
Semler et al. [10]	928/7942	975/7860	0.94 [0.87, 1.02]	0.94 [0.87, 1.02]
Summary	1015/8462	1058/8314	0.94 [0.87, 1.02]	0.94 [0.87, 1.02]
Fixed model heterogeneity: heterogeneity: *χ*^2^ = 0.04, *df* = 1 (*p* = 0.84); *I*^2^ = 0%; test for overall effect: *Z* = 1.51 (*p* = 0.13)				
Random model heterogeneity: *τ*^2^ = 0.00; *χ*^2^ = 0.04, *df* = 1 (*p* = 0.84); *I*^2^ = 0%; test for overall effect: *Z* = 1.52 (*p* = 0.13)				

Outcomes	Balanced crystalloids	0.9% Saline	Risk ratio/standard mean difference (95% CI)	
Development of stage 2 or higher AKI	Events/total	Events/total	Fixed models	Random models
Waters et al. [20]	4/33	5/33	0.80 [0.24, 2.72]	0.80 [0.24, 2.72]
Young et al. [23]	3/22	6/24	0.55 [0.15, 1.92]	0.55 [0.15, 1.92]
Young et al. [9]	105/1067	104/1025	0.97 [0.75, 1.25]	0.97 [0.75, 1.25]
Verma et al. [24]	1/33	3/34	0.34 [0.04, 3.14]	0.34 [0.04, 3.14]
Semler et al. [25]	97/520	87/454	0.97 [0.75, 1.26]	0.97 [0.75, 1.26]
Semler et al. [10]	807/7558	858/7458	0.93 [0.85, 1.02]	0.93 [0.85, 1.02]
Summary	1017/9233	1063/9028	0.93 [0.86, 1.01]	0.93 [0.86, 1.01]
Fixed model heterogeneity: *χ*^2^ = 1.74, *df* = 5 (*p* = 0.88); *I*^2^ = 0%; test for overall effect: *Z* = 1.71 (*p* = 0.09)				
Random model heterogeneity: *τ*^2^ = 0.00; *χ*^2^ = 1.74, *df* = 5 (*p* = 0.88); *I*^2^ = 0%; test for overall effect: *Z* = 1.69 (*p* = 0.09)				

Outcomes	Balanced crystalloids	0.9% Saline	Risk ratio/standard mean difference (95% CI)	
New RRT use	Events/total	Events/total	Fixed models	Random models
Takil et al. [21]	0/15	0/15	Not estimable	Not estimable
Young et al. [9]	38/1152	38/1110	0.96 [0.62, 1.50]	0.96 [0.62, 1.50]
Verma et al. [24]	5/33	3/34	1.72 [0.45, 6.62]	1.72 [0.45, 6.62]
Semler et al. [25]	24/520	14/454	1.50 [0.78, 2.86]	1.50 [0.78, 2.86]
Semler et al. [10]	189/7588	220/7458	0.84 [0.70, 1.02]	0.84 [0.70, 1.02]
Summary	256/9308	275/9071	0.91 [0.77, 1.07]	0.95 [0.75, 1.21]
Fixed model heterogeneity: *χ*^2^ = 3.77, *df* = 3 (*p* = 0.29); *I*^2^ = 20%; test for overall effect: *Z* = 1.16 (= 0.24)				
Random model heterogeneity: *τ*^2^ = 0.01; *χ*^2^ = 3.77, *df* = 3 (*p* = 0.29); *I*^2^ = 20%; test for overall effect: *Z* = 0.38 (*p* = 0.70)				

Outcomes	Balanced crystalloids	0.9% Saline	Risk ratio/standard mean difference (95% CI)	
RRT-free days	Mean ± SD, No.	Mean ± SD, No.	Fixed models	Random models
Semler et al. [25]	24.9 ± 9.7, 520	23.7 ± 10, 520	0.12 [− 0.00, 0.25]	0.12 [− 0.00, 0.25]
Semler et al. [10]	25.6 ± 8.6, 7942	24.8 ± 8.9, 7860	0.09 [0.06, 0.12]	0.09 [0.06, 0.12]
Summary			0.09 [0.06, 0.12]	0.09 [0.06, 0.12]
Fixed model heterogeneity: *χ*^2^ = 0.38, *df* = 1 (*p* = 0.54); *I*^2^ = 0%, test for overall effect: *Z* = 6.02 (*p* < 0.00001)				
Random model heterogeneity: *τ*^2^ = 0.00; *χ*^2^ = 0.21, *df* = 1 (*p* = 0.65); *I*^2^ = 0%; test for overall effect: *Z* = 6.03 (*p* < 0.00001)				

*CI* confident interval, *AKI* acute kidney injury, *RRT* renal replacement therapy, *SD* standard difference, *SMD* standard mean difference

**Fig. 3:**
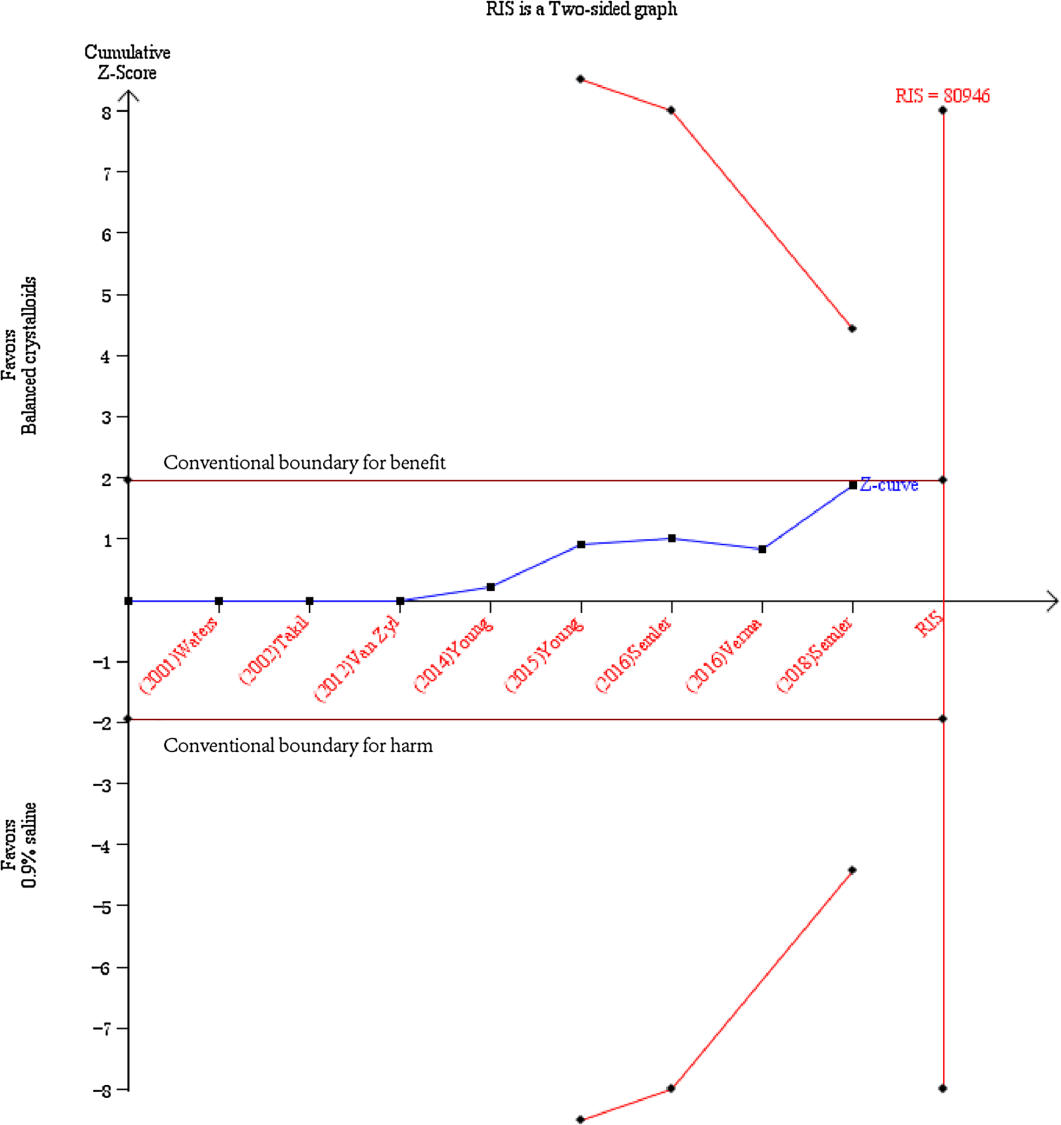
Trial sequential analysis for in-hospital mortality. TSA was performed based on a relative risk reduction of in-hospital mortality of 6.42% according to 10.2% in balanced crystalloids group and 10.9% in 0.9% saline group in eight trials with 19,301 patients reporting in-hospital mortality. A required diversity-adjusted information size of 80,946 patients was calculated. The cumulated *Z*-curve (blue) failed to reach the traditional boundary (*p* = 0.05), the trial sequential monitoring boundary as well as the estimated information size boundary. TSA is for trial sequential analysis

In the TSA shown in Fig. 3, a required diversity-adjusted information size of 80,946 patients was calculated with the relative risk reduction of 6.42% according to the in-hospital mortality of 10.2% and 10.9% in balanced crystalloids group and 0.9% saline group, respectively [9, 10, 20–25]. The cumulated *Z*-curve (blue) failed to reach the traditional boundary (*p* = 0.05), the trial sequential monitoring boundary as well as the estimated information size boundary (Fig. 3), indicating insufficient evidence to draw the conclusion.

Subgroup analysis revealed that the effect of balanced crystalloid fluid on in-hospital mortality was not associated with the balanced crystalloid fluid type, volume or patients’ settings (Table 3). No significant differences were detected in subpopulations with non-sepsis (5 trials, RR = 0.96; 95% CI, 0.86–1.07; *p* = 0.48, *I*^2^ = 0%, Table 3) or TBI (2 trials, RR = 1.11; 95% CI, 0.86–1.43; *p* = 0.02, *I*^2^ = 0%, Table 3) with balanced crystalloids administration compared with 0.9% saline. Of note, significant differences were observed in septic and non-traumatic patients.

**Table 3:** Effects of chloride content of intravenous crystalloid solutions on in-hospital mortality by subgroups

Subgroups	No. of studies	No. of patients	RR (M–H, random, 95% CI)	*p* value	*I*^2^ (%)
Crystalloid fluid type					
LR [20–22]	3	150	1.00 [0.07, 15.33]	1.00	NA
Plasma-Lyte [9, 23, 24]	3	2375	0.91 [0.70, 1.19]	0.50	0
Mixed of LR and Plasma-Lyte [10, 25]	2	16776	0.93 [0.85, 1.01]	0.08	0
Test for subgroup differences: *χ*^2^ = 0.01, *df* = 2 (*p* = 0.99)					
Crystalloid fluid volume					
Median or mean volume more than 5L [20, 21, 23]	3	142	0.86 [0.25, 2.94]	0.80	0
Median or mean volume less than 5L [9, 10, 24, 25]	4	19105	0.92 [0.85,1.00]	0.06	0
Unclear [22]	1	54	NA	NA	NA
Test for subgroup differences: *χ*^2^ = 0.01, *df* = 1 (*p* = 0.90)					
Setting					
ICU [9, 10, 22–25]	6	19205	0.92 [0.85,1.00]	0.06	0
Transferred to ICU in perioperative period [20, 21]	2	96	1.00 [0.07, 15.33]	1.0	NA
Test for subgroup differences: *χ*^2^ = 0.00, *df* = 1 (*p* = 0.95)					
*Specific patients*					
Sepsis					
Yes [9, 10]	2	2420	0.86 [0.75, 0.98]	0.02	0
No [9, 10, 20–22]	5	15794	0.96 [0.86, 1.07]	0.48	0
Classification not mentioned [23–25]	3	1087	0.95 [0.66, 1.37]	0.79	0
Test for subgroup differences: *χ*^2^ = 1.8, *df* = 2 (*p* = 0.41)					
TBI					
Yes [9, 10]	2	1420	1.11 [0.86, 1.43]	0.43	0
No [9, 10, 20–22]	5	16794	0.90 [0.82, 0.99]	0.02	0
Classification not mentioned [23–25]	3	1087	0.95 [0.66, 1.37]	0.79	0
Test for subgroup differences: *χ*^2^ = 2.31, *df* = 2 (*p* = 0.32)					
Risk of bias					
High [10, 23, 25]	3	16822	0.92 [0.85, 1.01]	0.98	0
Low [9, 20, 22, 24]	4	2449	0.91 [0.69, 1.20]	0.42	0
Unclear [21]	1	30	NA	NA	NA
Test for subgroup differences: *χ*^2^ = 0.01, *df* = 1 (*p* = 0.93)					
Including sample size (in each group)					
Over 1000 [9, 10]	2	18064	0.92 [0.85, 1.00]	0.06	0
Equal or less than 1000 [20–25]	6	1237	0.95 [0.71, 1.28]	0.75	0
Test for subgroup differences: *χ*^2^ = 0.05, *df* = 1 (*p* = 0.82)					

The random-effects model was applied above

*No.* number, *RR* risk ratio, *CI* confident interval, *NA* not applicable, *ICU* intensive care unit, *TBI* traumatic brain injury

**Fig. 4:**
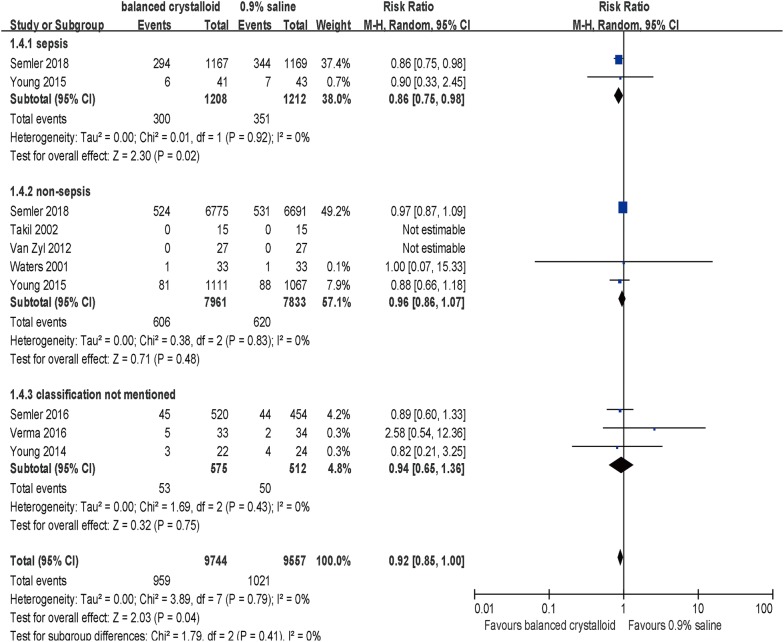
Forest plots for in-hospital mortality in subgroups of sepsis and non-sepsis

**Fig. 5:**
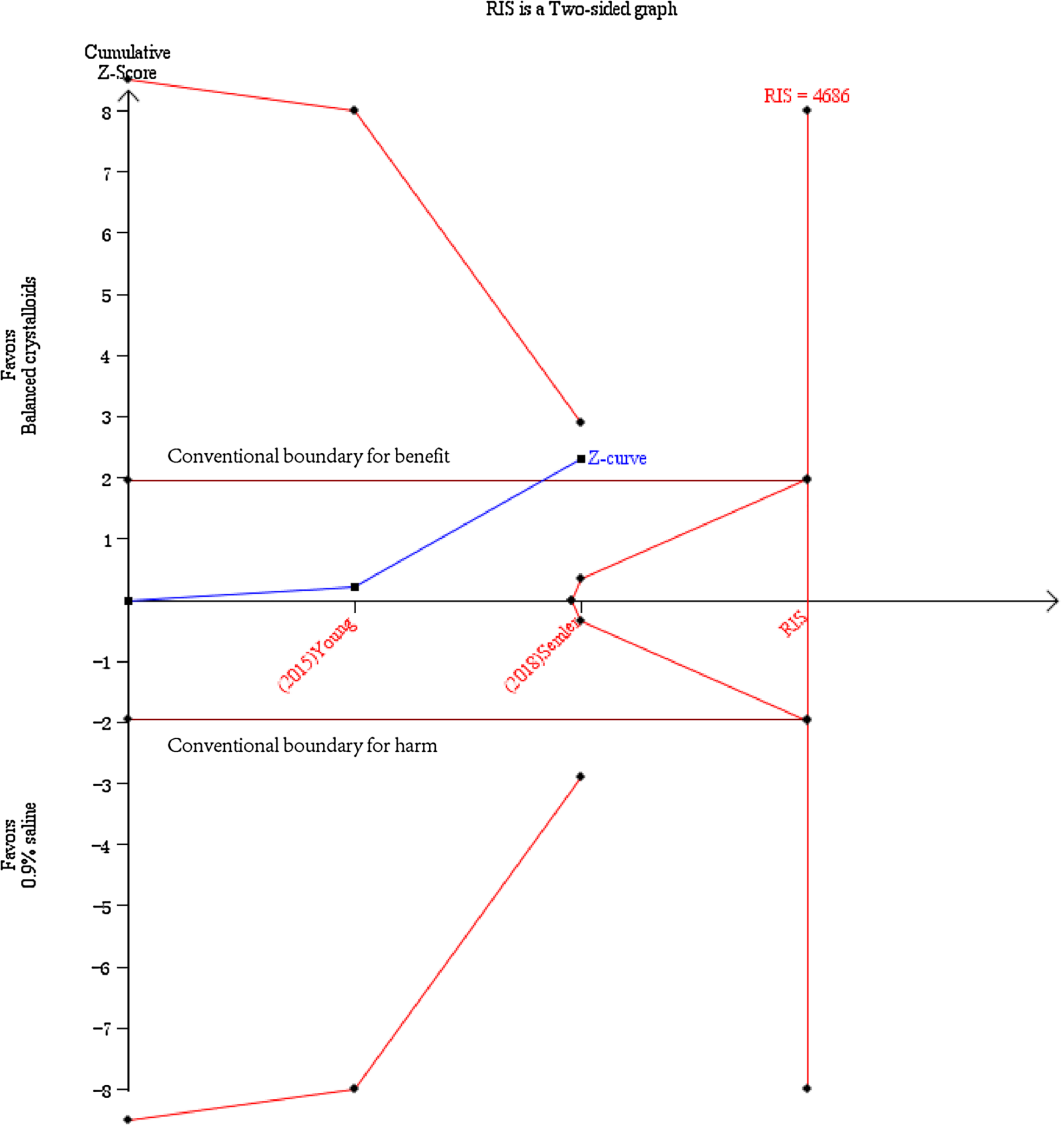
Trial sequential analysis for in-hospital mortality in subgroup of septic patients. A relative risk reduction relative risk reduction of 14.48% according to the in-hospital mortality in balanced crystalloids group (24.8%) and the control group (29%) was pursued. The cumulated *Z*-curve (blue) crossed the conventional boundary, but did not exceed either the trial sequential monitoring boundary for benefit or the required information size of 4686

Notably, a reduced in-hospital mortality rate was detected in subgroup analysis to be associated with balanced crystalloids compared with 0.9% saline among septic patients (*n* = 2420; RR = 0.86; 95% CI, 0.75–0.98; *p* = 0.02, *I*^2^ = 0%, Table 3) rather than non-septic patients (RR = 0.96; 95% CI, 0.86–1.07; *p* = 0.48, Table 3) despite no substantial evidence of such differences when trials were stratified by sepsis or not (*p* = 0.18 for interaction, Fig. 4). Besides, TSA was performed with expectation of relative risk reduction of 14.48% according to the in-hospital mortality in balanced crystalloids group (24.8%) and the control group (29%) [9, 10, 20–22]. The *Z*-curve crossed the conventional boundary, but did not exceed either the trial sequential monitoring boundary for benefit or the required information size of 4686, suggesting that this meta-analysis could not draw firm positive results for the insufficient sample currently (Fig. 5).

**Fig. 6:**
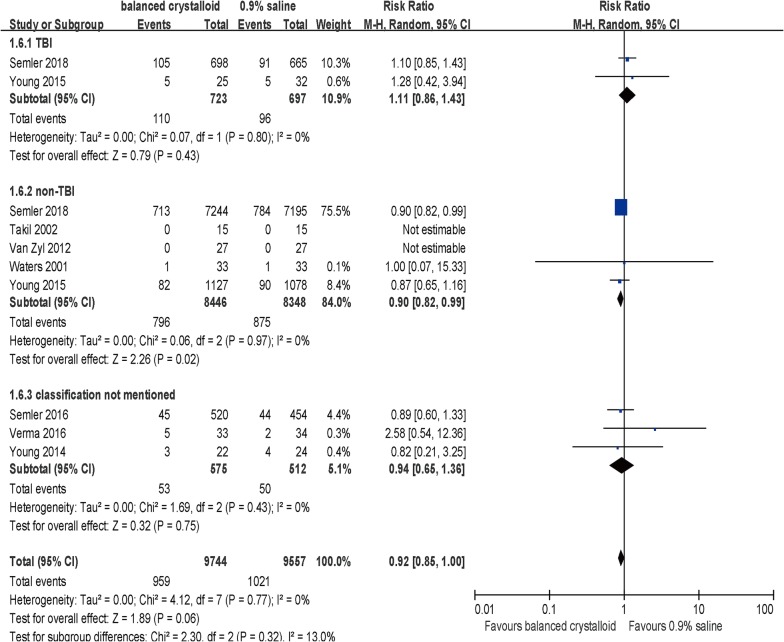
Forest plots for in-hospital mortality in subgroups of the traumatic brain injury (TBI) and non-TBI

**Fig. 7:**
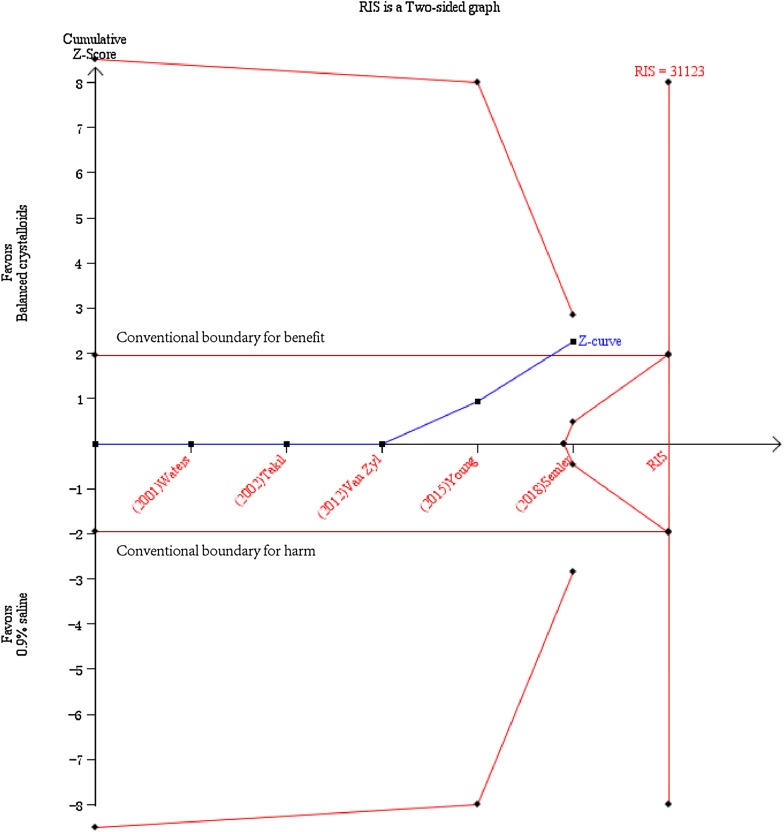
Trial sequential analysis for in-hospital mortality in subgroup of non-traumatic brain injury patients. Trial sequential analysis for a relative risk reduction of 10.48% according to the in-hospital mortality of 9.4% and 10.5% of patients without TBI in balanced crystalloids group and 0.9% saline group, respectively [9, 10, 20–22], was performed. A required diversity-adjusted information size of 31,123 patients was calculated. The cumulated *Z*-curve (blue) crossed the conventional line, but reached neither the trial sequential monitoring boundary for benefit nor the estimated information size boundary. TBI is for traumatic brain injury

Subgroup analysis revealed that balanced crystalloid fluid administration was associated with a lower in-hospital mortality rate when compared with 0.9% saline group for patients without traumatic brain injury (*n* = 16,794; RR = 0.90; 95% CI, 0.82–0.99; *p* = 0.02, Table 3), but not the patients with traumatic brain injury (*n* = 1420; RR = 1.11; 95% CI, 0.86–1.43; *p* = 0.43, Table 3). This is despite the fact that we found no substantial evidence of such differences when trials were stratified by TBI or not (*p* = 0.13 for interaction, Fig. 6). In the TSA, a required information size of 31,123 patients was calculated with the relative risk reduction of 10.48% according to the in-hospital mortality of 9.4% and 10.5% of patients without TBI in balanced crystalloids group and 0.9% saline group, respectively [9, 10, 20–22]. The *Z*-curve crossed the conventional line, but reached neither the trial sequential monitoring boundary for benefit nor the estimated information size boundary (Fig. 7), indicating lack of samples to reach the reliable and conclusive cumulative evidence.

### Impact on renal outcomes

Meta-analysis results for renal end points (receipt of new RRT, stage 2 or higher AKI development and RRT-free days) are summarized in Table 2. No statistically significant impact on development of stage 2 or higher AKI was detected in the three studies included (*I*^2^ = 0, *p* = 0.11) [9, 10, 25]. A total of 531 patients received new RRT after enrollment in the five trials included, and there was no apparent effect in patients treated with balanced crystalloid compared to 0.9% saline (*I*^2^ = 0%, *p* = 0.24). Among them, three studies (9, 10, 25) with 423 patients provided detailed indications for new RRT use, which included oliguria, acidemia, blood urea nitrogen over 70 mg/dL, serum creatinine over 3.39 mg/dL and organ edema. The data showed no difference between groups of patients treated with balanced crystalloid and 0.9% saline solutions (Additional file 1: Figure S3). However, regardless of no difference of new RRT use, there was a significant advantage with respect to longer RRT-free day in patients treated with balanced crystalloid solutions compared to the controls (SMD 0.09, 95% CI [0.06, 0.12], *I*^2^ = 0, *p* < 0.001, Table 2).

Two studies of Selmer in 2016 and 2018 introduced the composite outcome of MAKE30, which is defined as the occurrence of any of the following: in-hospital mortality, new RRT use or a 200% increase in serum creatinine from admission to discharge at 30 days after ICU admission [10, 25]. The effect of balanced crystalloid fluid administration seemed to be protective in reducing MAKE30 incidence for subgroups of patients with sepsis (RR = 0.81, 95% CI, 0.66–1.01, *p* = 0.06, Additional file 1: Fig S4a). Balanced crystalloid fluid administration was found to be associated with a reduction in incidence of MAKE30 in patients receiving RRT prior to enrollment when compared with the controls (RR = 0.73, 95% CI, 0.55–0.97, *p* = 0.03, Additional file 1: Fig S4b), while there was no difference among subgroups stratified according to baseline renal function (*p* = 0.31 for interaction).

### Impact on serum content

Three studies [20, 21, 23] provided data of biochemical values before and after crystalloid fluids administration (Additional file 1: Figure S5). Analysis of effects on serum content revealed that balanced crystalloid fluid was associated with significantly lower risk of increase in serum chloride (SMD − 1.23, 95% CI [− 1.59, − 0.87], *p* < 0.001; *I*^2^ = 0%) and sodium (SMD − 1.28, 95% CI [− 1.65, − 0.92], *p* < 0.001; *I*^2^ = 0%) compared to 0.9% saline. There was a significant decline in serum base deficit (SMD − 0.58, 95% CI [− 0.98, − 0.18], *p* = 0.004; *I*^2^ = 26%) with 0.9% saline compared to balanced crystalloid fluid infusion. There was no significant difference in alterations of serum pH value and bicarbonate concentration between balanced crystalloids and 0.9% saline groups.

### Impact on organ function

There was no significant difference between balanced crystalloids versus 0.9% saline with respect to use of MV, but balanced crystalloid fluid was associated with significantly longer ventilator-free days (SMD 0.08, 95% CI [0.05, 0.11], *p* < 0.001; *I*^2^ = 0%) and vasopressor-free days (SMD 0.04, 95% CI [0.00, 0.07], *p* = 0.02; *I*^2^ = 0%, Additional file 1: Figure S6) compared to the control.

## Discussion

Consist with the previous meta-analysis [11, 26], the available RCTs fail to prove significant difference in in-hospital mortality and show a slight trend of in-hospital survival benefit without statistic difference toward balanced crystalloid solutions administration compared with 0.9% saline in critically ill adult patients. However, we found that assignment to balanced crystalloid solutions instead of 0.9% saline was associated with reduced mortality risk among the subgroup of patients with sepsis or non-TBI despite insufficient samples within present trials to draw the robust conclusion. In addition, the use of balanced crystalloid solutions was associated with longer RRT-free days, MV-free days and vasopressor-free days as well as less risk of increase in serum concentrations of chloride and sodium and less risk of decline in serum base deficit.

The use of 0.9% saline was proved with risk of hyperchloremic metabolic acidosis and related complications [7], suggesting an increase in death risk. Our meta-analysis revealed no statistically beneficial effect of balanced crystalloid fluid on in-hospital survival in critically ill adult patients compared with 0.9% saline. The neutral result might be explained by the following reasons. Firstly, an optimal sample size was not achieved according to the TSA results for reaching the reliable and conclusive cumulative evidence. Besides, the clinical heterogeneity should be taken into considerations. As the Semler study in 2016 suggested, there was a “dose-dependent” relationship between the development of major adverse kidney event and volume of crystalloid fluid received [25]. The relatively low volume of fluid exposure in selected study (median volume of 4 trials is no more than 5 L) during follow-up period [9, 10, 24, 25] may underestimate the effects of crystalloid fluid on prognosis. Thirdly, most patients included in our meta-analysis held a relatively low death risk of 10.1% (986/9744) and 10.9% (1045/9557) in groups of balanced crystalloids and 0.9% saline, respectively. The potential harm might surface among patients with high risk of death and renal injury. In this line, the effects of crystalloids administration might be underestimated by such a small proportion of included patients at high risk of acute kidney injury or who required large volumes of intravenous fluids, such as septic or septic shock patients. Hence, we conducted subgroup analyses by volume and type of crystalloid fluid, patient settings as well as specific patients with sepsis or not, TBI or not and different baseline of renal function. Of note, the difference in reducing in-hospital mortality between balanced crystalloids and saline appeared to be greater for patients with sepsis and non-traumatic brain injury.

For sepsis and septic shock, fluid therapy remains the cornerstone of hemodynamic resuscitation [27, 28]. Septic patients were likely to need larger volume of crystalloid fluid than those of non-sepsis and held higher severity of disease with elevated risk of renal injury and death. In our study, the calculated death risk in groups of balanced crystalloids and 0.9% saline was 24.8% (300/1208) and 29.0% (351/1212), respectively, in septic subpopulations, higher than 10.1% (986/9744) and 10.9% (1045/9557) for overall population. The subgroup analysis showed balanced crystalloid fluid administration could benefit septic patients with significantly reduced death risk, in accordance with the 2018 Selmer study and parts of review by Rochwerg in 2014 [8, 10]. However, there was no significant difference in the subgroup analysis stratified by the volumes of crystalloids fluid administration, which might be explained by the bias following the quite small population of 142 in group receiving larger volume of 5 L leading to a large variation. More samples were still in need to draw the firm positive results for septic patients according to the TSA. These findings suggest that it is necessary to be aware of the risks to septic patients in clinical management of fluid resuscitation.

Subgroup analysis and TSA revealed trends toward accumulative evidence that patients without TBI would benefit from the use of balanced crystalloid fluid. Previous studies have recognized that 0.9% saline solutions could benefit the TBI patients from reduced complications of cerebral edema and intracranial hypertension with a relatively higher osmolality [29–31]. The use of balanced crystalloid fluid may worsen the condition of TBI patients for relatively low osmolality with risk of cerebral edema and intracranial hypertension [32], which is clinically important.

It is worth noticing that two large studies of SPLIT [9] and SMART [10] trials consist of more than 98% of patients. Our meta-analysis showed similar results with SPLIT (9) and SMART (10) studies regarding to comparisons between balanced crystalloid fluid versus 0.9% saline on in-hospital mortality of critically ill adult patients. There was no effect on statistical results either following variety of sample size in subgroup analysis or when removing each single study in sensitivity analysis. The evidence levels remain debatable though results of large-scale RCTs seemed persuasive. The results did not differ among studies of high, low or unclear risk levels as subgroup analysis shown in subgroup analysis, while the Semler study in 2018 was judged to be at low risk of bias due to performance bias, degrading the level of evidence despite contributing the largest sample size.

Inconsistent with the before and after treatment study in which balanced crystalloid solutions administration was associated with decreasing the incidence of AKI and RRT use [7], our meta-analysis showed no difference between balanced crystalloid and 0.9% saline fluid groups regarding development of stage 2 or higher AKI and new RRT use. Our study found statistically longer RRT-free days with use of balanced crystalloid fluid in the two selected trials [10, 25], despite no statistical difference regarding incidence of RRT use compared to 0.9% saline. Besides, after stratifying the patients by categories of baseline kidney function, we found that balanced crystalloid fluid was associated with decreased risk of the incidence of MAKE30 in prior RRT subgroup [10, 25]. Therefore, further studies remain in need to make meaningful comparisons in terms of renal end points for heterogeneous ICU populations with categories of baseline kidney function.

Our findings showed that balanced crystalloid fluid administration was associated with less increase in serum concentrations of chloride and sodium and less decline in serum base deficit, which is in agreement with previous literature data [33]. Of note, the data in our study showed the alterations of serum content without considerations of preconditions such as hypernatremia/hyponatremia or hyperchloremia/hypochloremia, in which effects of balance crystalloid fluid might differ between trials.

Our study merges several strengths. Two of the largest RCT trials, SMART [10] and SPLIT trials [9], were included with which the total sample exceeded 19,000. We believe that our study is one of the first meta-analyses with TSA to assess the effects of chloride content of intravenous crystalloid fluid on critically ill patients. We did an estimation of optimal sample size to provide the definite conclusion, which was not performed in Zayed’s review published recently [26]. What is more, our predefined subgroups took clinical heterogeneity into consideration and results of subgroup analyses provide a trend toward accumulative evidence of benefit in use of balanced crystalloid fluid in septic and non-TBI populations in ICU. These findings provide important and reasonable suggestions between the fluid types for clinical management for patients with sepsis or non-traumatic brain injury.

There are several limitations in our study. First, the evidence was limited for the following reasons. There were variable risks of performance and attrition bias of the included trials as described above, downgrading the quality of the evidence. Despite the statistically low heterogeneity and inconsistency by M–H Chi-square test and the *I*^2^ test, the potential heterogeneity originating from varied duration of follow-up and fluid exposure could not be ignored. TSA confirmed no firm conclusion of the results. Accordingly, a larger population with better designed would add more power to the results. Above all, we concluded that limited evidence supported statistical survival benefit with balanced crystalloid solutions according to the GRADE system, which was consistent with the previous literature. Second, rather than relatively small sample size studies, the potential bias following the two large studies of SMART and SPLIT trials with more than 98% of patients could not be ignored despite no statistical difference according to sensitivity analysis and subgroup analysis. Third, although subgroup analyses were performed, heterogeneity existed among trials included. Subgroups in terms of volume exposure were predefined according to median and mean value without considering the range and variance, which might influence results when pooling data. In addition, for the studies presented here, the volume of crystalloid fluid varied from less than 2 L to above 10 L with variable follow-up and incomparable data of daily fluid intake, which limited further analysis of its confounded impact on patients’ outcomes. We estimated the change of serum content before and after the use of crystalloid fluid according to recordable mean and standard deviation as the transferring formulas published. The further discussion regarding effects of balanced crystalloid fluid could not be performed in lack of baseline as mentioned above. The pragmatic ongoing trials [34, 35] may provide high-quality evidence on whether a low-chloride balanced crystalloid, compared with 0.9% saline, improves important clinical outcomes in critically ill patients.

## Conclusions

Longer free days of organ support and less fluctuations of serum electrolyte levels as well as base deficit were found with use of balanced crystalloid solutions. Current evidence is insufficient to draw a firm conclusion on mortality with a slight trend of survival benefit toward balanced crystalloid solutions. Nevertheless, our subgroup analysis suggested that its administration was associated with a decrease in in-hospital mortality in septic and non-TBI patients. Large-scale rigorous randomized trials with relatively large fluid exposure among patients of high risk are needed to provide robust evidence for guiding crystalloid fluid choice in critically ill patients.

## Additional file

**Additional file 1: Table S1.** Sensitivity analysis of the effects of chloride content in crystalloid fluid on critically ill patients’ outcomes. **Figure S1**. **a** Risk of bias graph. Review authors’ judgements about each risk of bias item resented as percentages across all included studies. **b** Risk of bias summary for each included study Red (-) indicates high risk of bias; yellow (?) indicates unclear risk; and green (+) indicates low risk of bias. **Figure S2**. GRADE profile for assessing quality of evidence. **Figure S3**. Forest plots for indications of new RRT use after enrollment. **Figure S4**. Forest plots for MAKE30 in predefined subgroups. **a** Sepsis and non-sepsis subgroups. **b** Subgroups according to categories of baseline renal function. MAKE30 is for major adverse kidney events within 30 days. **Figure S5**. Forest plots for alterations’ of serum content among critically ill patients. **Figure S6**. Forest plots for organ support. **a** MV use of critically ill patients. **b** Ventilator-free day of critically ill patients. **c** Vasopressor-free days of critically ill patients. MV is for mechanic ventilation. **Figure S7**. Funnel plots. **a** Funnel plots for in-hospital mortality. **b** Funnel plots for 30-day mortality. **c** Funnel plots for 60-day mortality. **d** Funnel plots for development of stage 2 of higher AKI of critically ill patients. **e** Funnel plots for new RRT use of critically ill patients. **f** Funnel plots of for RRT-free days of critically ill patients. AKI is for acute kidney injury according to KDIGO criterion; RRT is for renal replacement therapy.

## Abbreviations

AKI: acute kidney injury
AKIN: acute kidney injury network
CI: confidence interval
CKD: chronic kidney disease
GRADE: grading of recommendations assessment, development and evaluation
KDIGO: kidney disease: improving global outcomes
MAKE30: major adverse kidney event within 30 days
MV: mechanical ventilation
M–H: Mantel–Haenszel
NS: normal saline
RCT: randomized controlled trial
RIFLE: risk, injury, failure, loss, and end-stage
RR: risk ratio
RRT: renal replacement therapy
SMD: standard mean difference
SSC: surviving sepsis campaign
TBI: traumatic brain injury
TSA: trial sequential analysis
