# Supplementary material for: Effects of chloride content of intravenous crystalloid solutions in critically ill adult patients: a meta-analysis with trial sequential analysis of randomized trials
Source: Ann Intensive Care. 2019 Feb 13;9:30. doi: 10.1186/s13613-019-0506-y (PMC6374495; doi:10.1186/s13613-019-0506-y)
Supplement: Supplementary file 1 — Additional file 1: Table S1. Sensitivity analysis of the effects of chloride content in crystalloid fluid on critically ill patients’ outcomes. Figure S1. a Risk of bias graph. Review authors’ judgements about each risk of bias item resented as percentages across all included studies. b Risk of bias summary for each included study Red (-) indicates high risk of bias; yellow (?) indicates unclear risk; and green (+) indicates low risk of bias. Figure S2. GRADE profile for assessing quality of evidence. Figure S3. Forest plots for indications of new RRT use after enrollment. Figure S4. Forest plots for MAKE30 in predefined subgroups. a Sepsis and non-sepsis subgroups. b Subgroups according to categories of baseline renal function. MAKE30 is for major adverse kidney events within 30 days. Figure S5. Forest plots for alterations’ of serum content among critically ill patients. Figure S6. Forest plots for organ support. a MV use of critically ill patients. b Ventilator-free day of critically ill patients. c Vasopressor-free days of critically ill patients. MV is for mechanic ventilation. Figure S7. Funnel plots. a Funnel plots for in-hospital mortality. b Funnel plots for 30-day mortality. c Funnel plots for 60-day mortality. d Funnel plots for development of stage 2 of higher AKI of critically ill patients. e Funnel plots for new RRT use of critically ill patients. f Funnel plots of for RRT-free days of critically ill patients. AKI is for acute kidney injury according to KDIGO criterion; RRT is for renal replacement therapy. [file 13613_2019_506_MOESM1_ESM.docx]

Pubmed Search ((("Plasma-lyte 148"[Supplementary Concept] OR "plasma-lyte" OR Plasmalyte OR "ringer lactate" OR Ringer* OR "ringers lactate" OR "Balanced solution" OR "balanced saline" OR low chloride OR buffered crystalloid OR buffered solution))) AND ("Sodium Chloride"[Mesh] OR Saline OR NaCl 0.9%) AND ((randomized controlled trial[pt] OR controlled clinical trial[pt] OR randomized[tiab] OR placebo[tiab] OR drug therapy[sh] OR randomly[tiab] OR trial[tiab] OR groups[tiab] NOT (animals[mh] NOT humans[mh])))

| **Table S1. Sensitivity analysis of the effects of chloride content in crystalloid fluid on critically ill patients’ outcomes** | | | |
| --- | --- | --- | --- |
| **Outcomes** | **Estimate** | **[95% Conf. Interval]** | |
| **In-hospital mortality** |  |  | |
| Waters et al, 2001 | 0.92 | 0.85 | 1.00 |
| Takil et al, 2002 | 0.92 | 0.85 | 1.00 |
| Van Zyl et al, 2012 | 0.92 | 0.85 | 1.00 |
| Young et al, 2014 | 0.92 | 0.85 | 1.00 |
| Young et al, 2015 | 0.93 | 0.85 | 1.01 |
| Verma et al, 2016 | 0.92 | 0.85 | 1.00 |
| Semler et al, 2016^a^ | 0.92 | 0.85 | 1.01 |
| Semler et al, 2018^b^ | 0.92 | 0.75 | 1.12 |
| **30-day mortality** |  |  |  |
| Young et al, 2014 | 0.93 | 0.85 | 1.01 |
| Semler et al, 2016 | 0.92 | 0.85 | 1.01 |
| Semler et al, 2018 | 0.92 | 0.68 | 1.23 |
| **60-day mortality** |  |  |  |
| Semler et al, 2016 | 0.94 | 0.87 | 1.02 |
| Semler et al, 2018 | 0.92 | 0.70 | 1.20 |
| **Development of stage 2 or higher AKI^c^** | | | |
| Semler et al, 2016 | 0.93 | 0.86 | 1.02 |
| Semler et al, 2018 | 0.97 | 0.81 | 1.17 |
| Young et al, 2015 | 0.93 | 0.86 | 1.02 |
| **New RRT use** |  |  |  |
| Takil et al, 2002 | 0.90 | 0.77 | 1.07 |
| Young et al, 2015 | 0.90 | 0.75 | 1.07 |
| Verma et al, 2016 | 0.90 | 0.76 | 1.06 |
| Semler et al, 2016 | 0.87 | 0.73 | 1.03 |
| Semler et al, 2018 | 1.14 | 0.80 | 1.62 |
| **RRT-free days** |  |  |  |
| Semler et al, 2016 | 0.94 | 0.87 | 1.02 |
| Semler et al, 2018 | 0.92 | 0.70 | 1.20 |

Conf. is for confidence; AKI is for acute kidney injury; RRT is for renal replacement therapy;

^a^The study of Semler in 2016 provided mortality of 30-day and 60-day. The 30-day mortality was used for in-hospital mortality；

^b^The study of Semler in 2018 provided mortality of 30-day and 60-day. The 30-day mortality was used for in-hospital mortality；

^c^according to Kidney Disease: Improving Global Outcomes(KDIGO) criterion.

**a.**


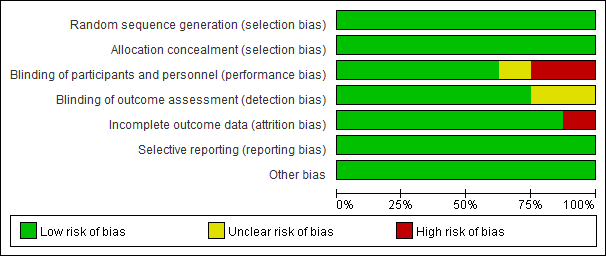


**b.**


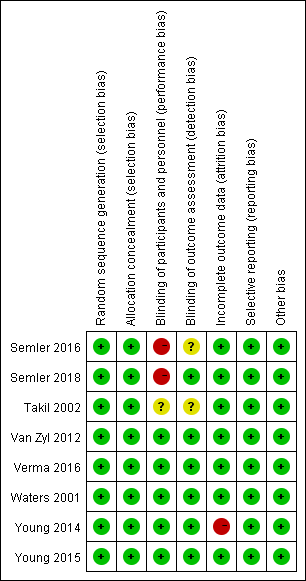


**Figure S1. a.** Risk of bias graph. Review authors’ judgements about each risk of bias item resented as percentages across all included studies. **b**. Risk of bias summary for each included study Red (-) indicates high risk of bias; yellow (?) indicates unclear risk; and green (+) indicates low risk of bias.


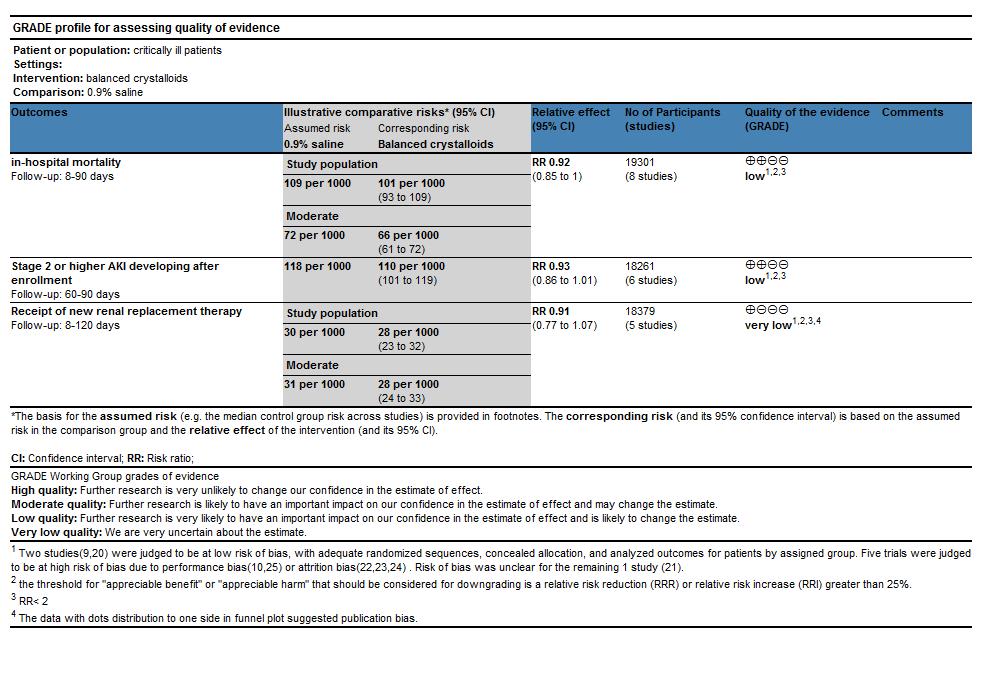


**Figure S2.** GRADE profile for assessing quality of evidence.

**
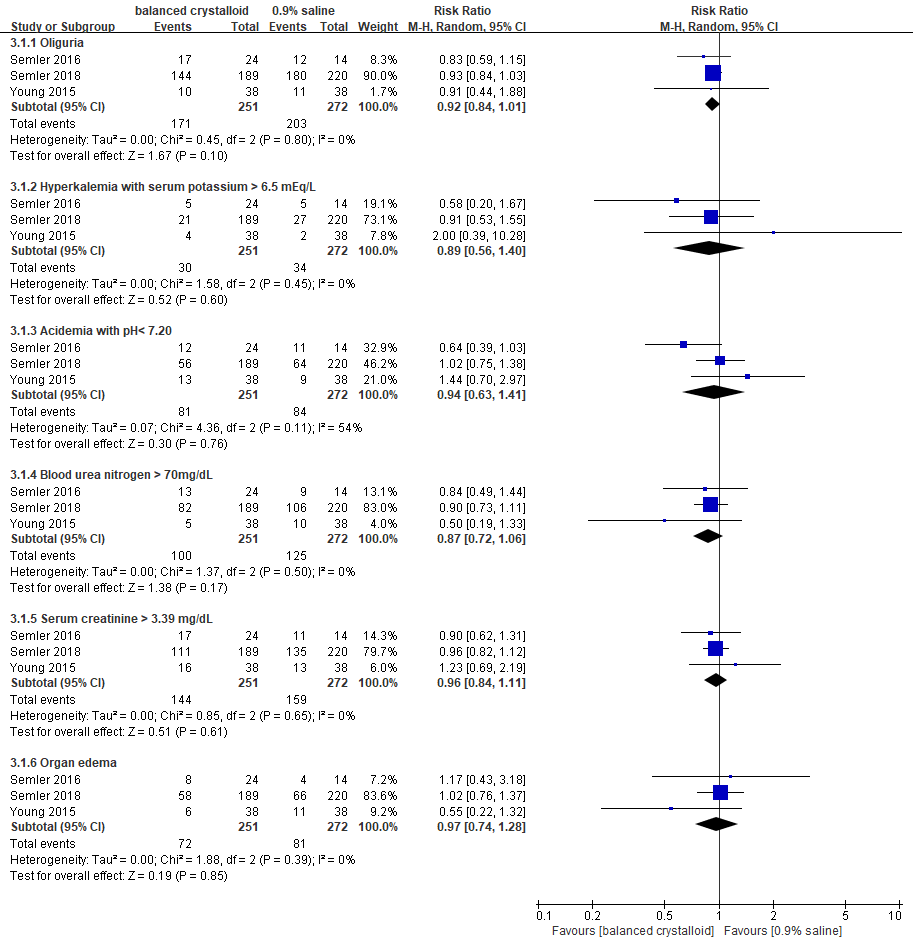
**

**Figure S3.** Forest plots for indications of new RRT use after enrollment.


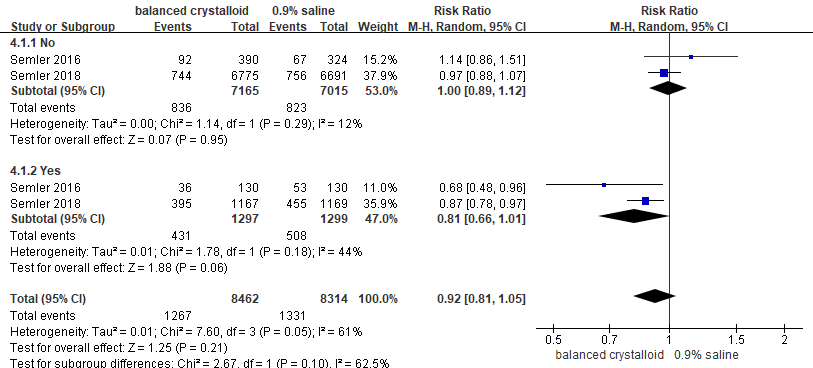
a. Sepsis or not


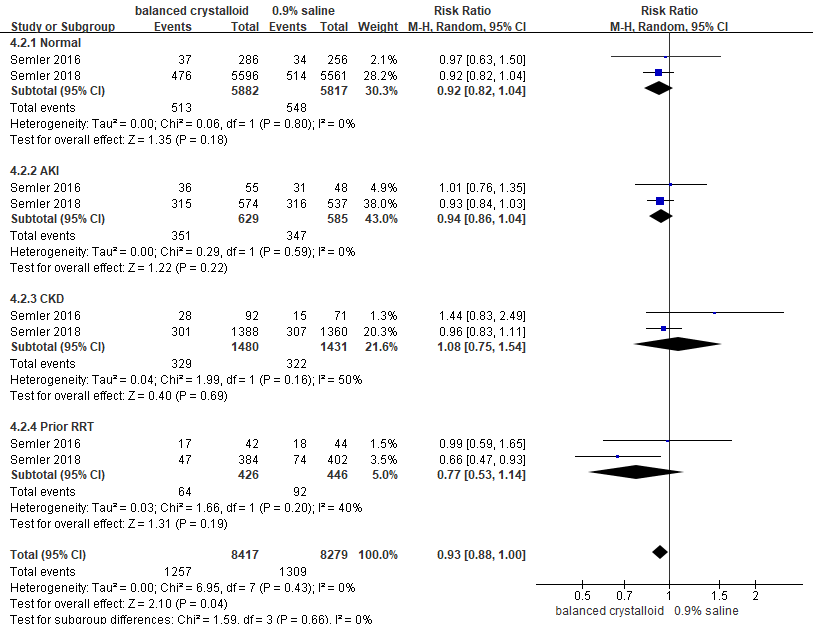
b. Baseline renal function

**Figure S4.** Forest plots for MAKE30 in predefined subgroups. **a.** Sepsis and non-sepsis subgroups. **b.** Subgroups according to categories of baseline renal function. MAKE30 is for major adverse kidney events within 30 days;


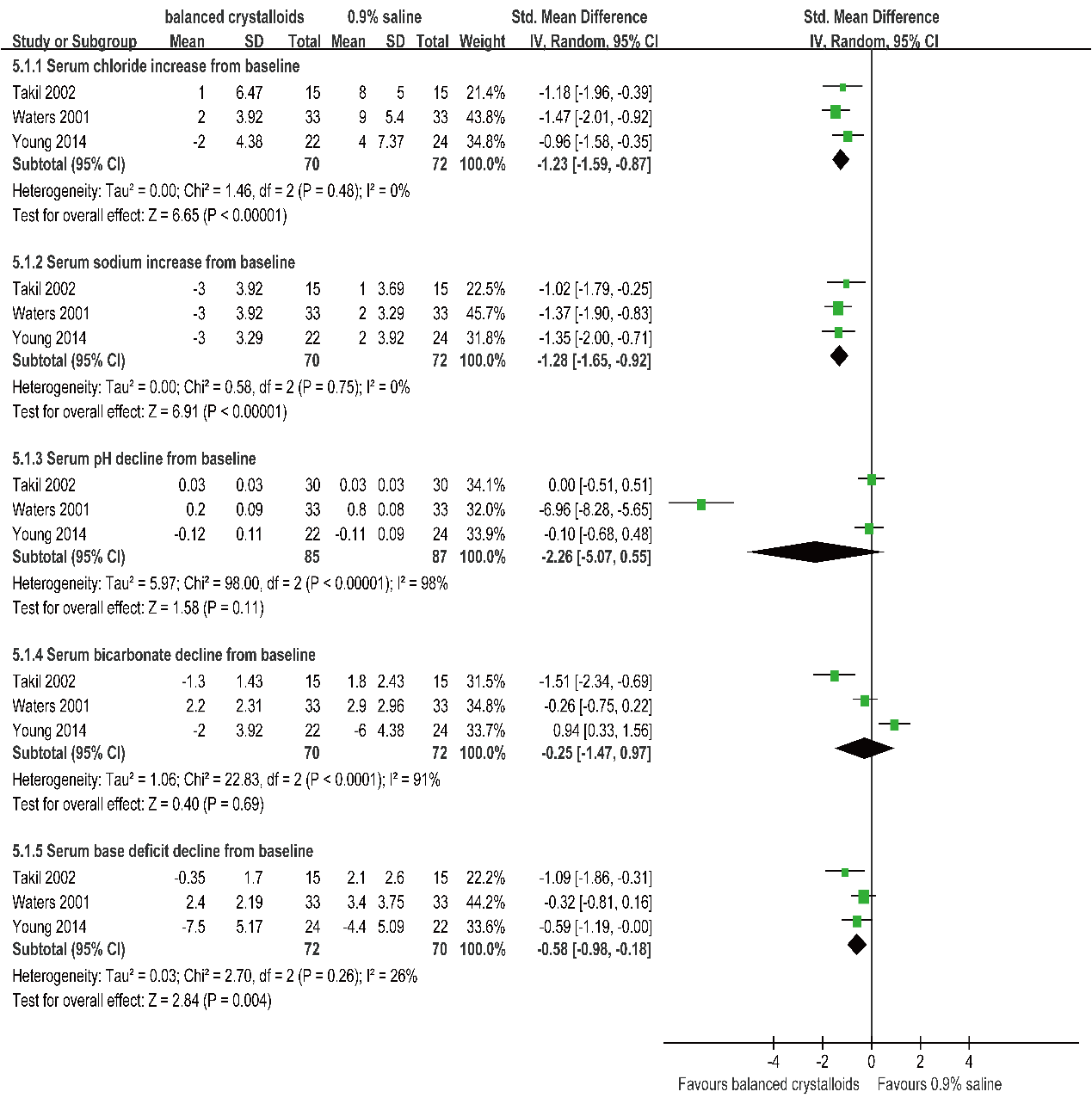


**Figure S5.** Forest plots for alterations’ of serum content among critically ill patients.


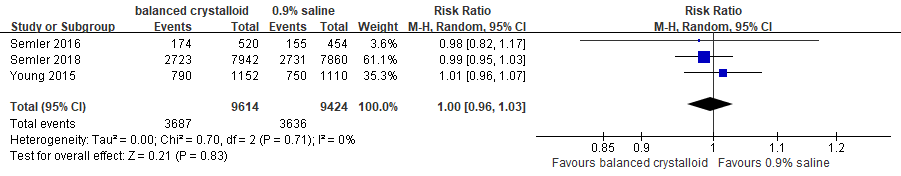
a. Use of mechanic ventilation


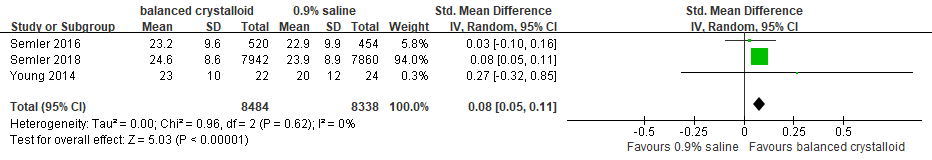
b. Ventilator-free days


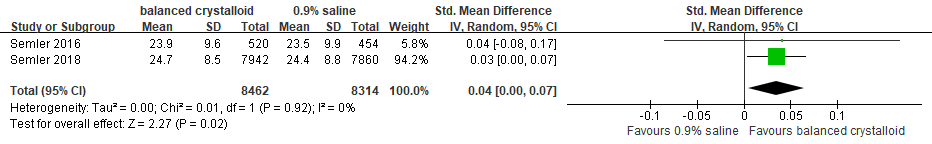
c. Vasopressor-free days

**Figure S6.** Forest plots for organ support. **a**. MV use of critically ill patients. **b.** Ventilator-free day of critically ill patients. **c.** Vasopressor-free days of critically ill patients. MV is for mechanic ventilation.


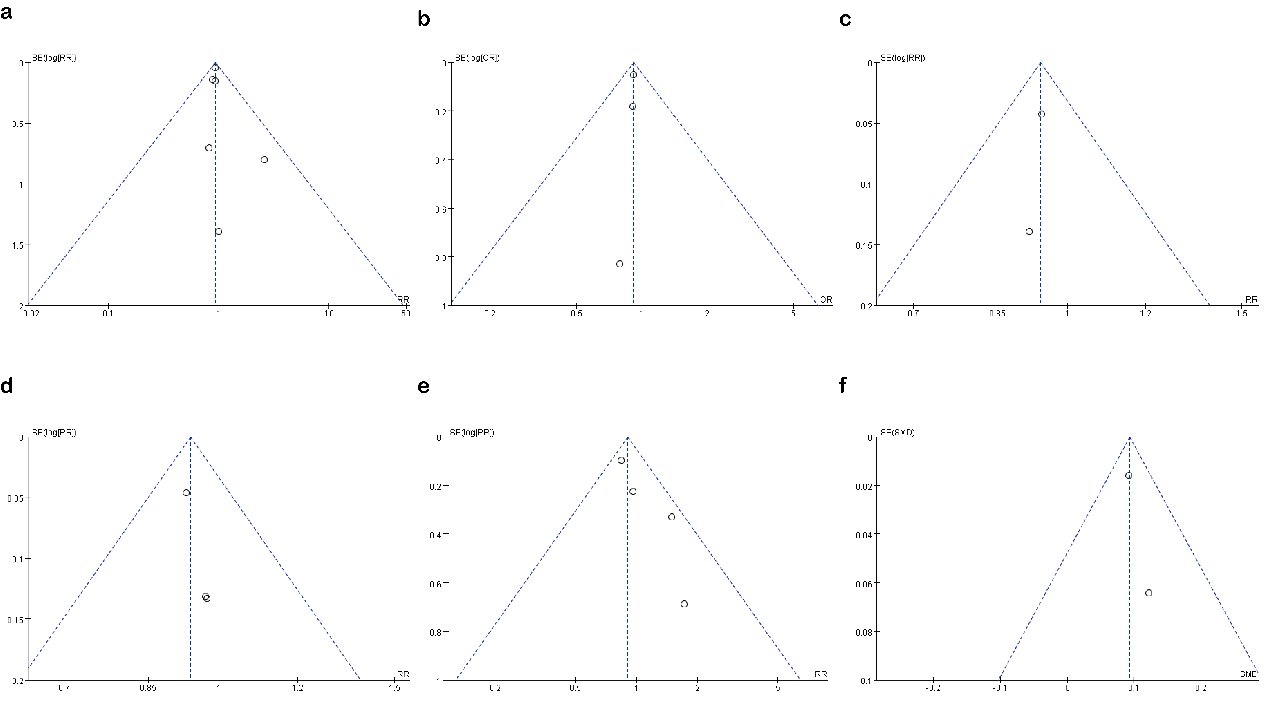


**Figure S7.** Funnel plots. **a**. Funnel plots for in-hospital mortality. **b**. Funnel plotsfor 30-day mortality. **c**. Funnel plots for 60-day mortality. **d**. Funnel plots for development of stage 2 of higher AKI of critically ill patients. **e**. Funnel plots for new RRT use of critically ill patients. **f**. Funnel plots of for RRT-free days of critically ill patients. AKI is for acute kidney injury according to KDIGO criterion; RRT is for renal replacement therapy.
